# Single-cell RNA sequencing reveals a reprogramming of hepatic immune cells and a protective role for B cells in MASH-driven HCC

**DOI:** 10.1097/HC9.0000000000000668

**Published:** 2025-04-21

**Authors:** Haiguang Wang, Adam Herman, Fanta Barrow, Amal Abdel-Ghani, Micah Draxler, Gavin Fredrickson, Preethy Parthiban, Davis M. Seelig, Sayeed Ikramuddin, Xavier S. Revelo

**Affiliations:** 1 Department of Integrative Biology and Physiology, University of Minnesota Medical School, Minneapolis, Minnesota, USA; 2 Center for Immunology, University of Minnesota, Minneapolis, Minnesota, USA; 3 Department of Surgery, University of Minnesota Medical School, Minneapolis, Minnesota, USA; 4 Veterinary Clinical Sciences Department, Comparative Pathology Shared Resource, University of Minnesota, Saint Paul, Minnesota, USA; 5 Institute for the Biology of Aging and Metabolism, University of Minnesota, Minneapolis, Minnesota, USA

**Keywords:** B cells, immune landscape, liver cancer, macrophages, T cells

## Abstract

**Background:**

HCC, the most common form of liver cancer, is one of the leading causes of cancer-related deaths worldwide. Although the immune system plays a crucial role in liver cancer pathogenesis, the immune landscape within metabolic dysfunction–associated steatohepatitis–driven HCC remains poorly understood.

**Methods:**

In this study, we used the high-fat, high-carbohydrate diet fed major urinary protein–urokinase-type plasminogen activator mouse model of metabolic dysfunction–associated steatohepatitis–driven HCC. We performed single-cell RNA sequencing on intrahepatic immune cells to characterize their heterogeneity and gene expression profiles. Additionally, we examined the role of B cells in antitumor immunity by depleting B cells in μMT mice and analyzing the effects on liver cancer progression.

**Results:**

Our analysis revealed significant shifts in intrahepatic immune cell populations, including B cells, T cells, and macrophages that undergo transcriptional reprogramming, suggesting altered roles in tumor immunity. Notably, an expanded subset of activated B cells in HCC mice showed an antitumor B cell gene expression signature associated with increased survival of patients with liver cancer. Consistently, B cell-deficient mice showed exacerbated liver cancer progression, a substantial reduction in intrahepatic lymphocytes, and impaired CD8^+^ T cell activation, suggesting that intrahepatic B cells may promote antitumor immunity by enhancing T cell responses.

**Conclusions:**

Our findings reveal a complex immune reprogramming within the metabolic dysfunction–associated steatohepatitis–driven HCC microenvironment and underscore a protective role for B cells in liver cancer. These results highlight B cells as potential targets for immunomodulatory therapies in HCC.

## Graphical abstract

**Figure:**
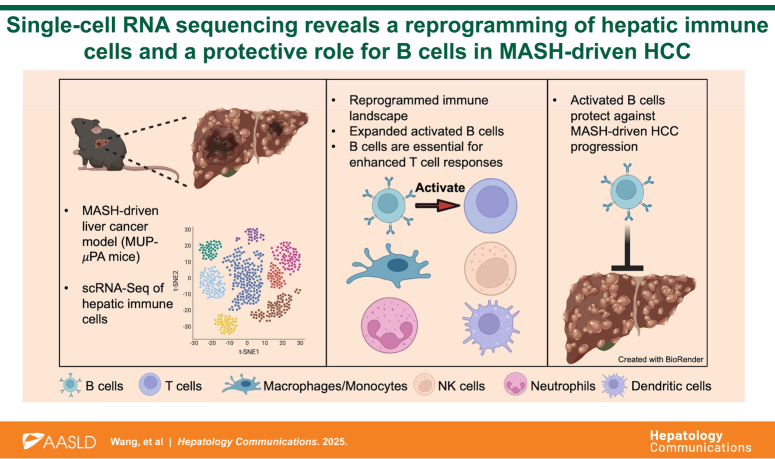

## INTRODUCTION

Primary and metastatic liver cancers are the leading causes of disease-related mortality.^1^ HCC is the most prevalent form of liver cancer^2^ and is projected to increase significantly by 2030.^3^ HCC has a poor prognosis due to the lack of effective treatments.^2^ Moreover, HCC is strongly associated with metabolic dysfunction–associated steatohepatitis (MASH), the major risk factor for HCC.^3^ Notably, the incidence of MASH-related HCC has increased nearly 8-fold, from ~2% to 16%.^4^ MASH is a severe progression of metabolic dysfunction–associated steatotic liver disease (MASLD),^5^ which affects about a quarter of the global population.^3^,^5^ Although the mechanisms by which MASH progresses to HCC are unclear, up to 12% of patients with MASH develop liver cancer.^6^ Murine studies suggest that MASH-associated inflammatory cytokines^7^ and dysregulated adaptive immune responses involving CD8^+^ cytotoxic T cells and IgA plasma cells^8^ contribute to HCC development. However, our understanding of how immune cells regulate the pathogenesis of MASH-driven HCC remains limited.

The immune system has a critical role in cancer progression, either combatting or facilitating tumorigenesis.^9^ Cytotoxic CD8^+^ T cells are central to antitumor immunity by directly targeting cancer cells,^10^,^11^ but T cell exhaustion can diminish their effectiveness.^10^ CD4^+^ T cells, including T_H_17 cells that produce IL-17, can also enhance cytotoxic responses and recruit immune cells.^12^ However, T_H_17 cells can also promote tumor growth and metastasis under certain conditions.^13^ In HCC, IL-17-producing cells have been associated with poor prognosis.^14^ B cells regulate antitumor immune responses through antibody-mediated cytotoxicity, antigen presentation, and immune regulation.^15^ B cells present tumor-associated antigens to T cells and produce antibodies that facilitate antigen presentation or directly eliminate tumor cells.^15^ Conversely, the formation of antibody–antigen immune complexes can promote inflammation, angiogenesis, and immunosuppression through the activation of macrophages and the complement system, leading to adverse effects.^16^ Dendritic cells (DCs) bridge innate and adaptive responses by presenting tumor antigens to T cells,^17^ but can become dysregulated within the tumor microenvironment, aiding immune evasion.^17^ Tumor-associated macrophages (TAMs) are generally pro-tumorigenic as they promote angiogenesis and facilitate tumor invasion and metastasis.^18^ However, TAMs can also be reprogrammed to be antitumoral.^19^ Neutrophils and innate lymphoid cells, including natural killer (NK) cells, are implicated in tumor surveillance.^20^,^21^ The plasticity of immune cell functions allows them to either inhibit or promote tumors, depending on the microenvironmental cues.^20^,^21^ Thus, a better understanding of the intrahepatic immune cells is essential for developing effective therapies for MASH-driven HCC.

Although several preclinical models of MASH-driven HCC exist, they often do not accurately resemble human disease. Transgenic mice expressing urokinase-type plasminogen activator (uPA) controlled by the major urinary protein (MUP) promoter in hepatocytes develop chronic endoplasmic reticulum stress.^22^ When fed a high-caloric diet, MUP-uPA mice show increased liver damage and inflammation, mirroring the progression from MASH to HCC.^7^,^8^,^23^ In this study, we analyzed the population dynamics and transcriptional reprogramming of intrahepatic immune cells in HCC livers using the MUP-uPA mouse model. Using single-cell RNA sequencing (scRNA-seq), we identified intricate reprogramming of the immune cell landscape, including alterations toward inflammatory responses. Our findings reveal a critical role of B cells in resisting tumor development. Overall, our study offers novel insights into the role of immune cell subsets in MASH-driven HCC development.

## METHODS

### Animals

Wild-type (Wt) C57BL/6J (000664) and μMT (B6.129S2-Ighm^tm1Cgn^/J, 002288) mice were purchased from The Jackson Laboratory. MUP-uPA mice were kindly provided by Dr Michael Karin at the University of California San Diego. At 6 weeks of age, mice were continued on a normal chow diet (NCD) or switched to a high-fat, high-carbohydrate diet (HFHC; 40% kcal palm oil, 20% kcal fructose, and 2% cholesterol) supplemented with 42 g/L of carbohydrates in the drinking water (55% fructose, 45% sucrose).^24^ All mice were male, age-matched, and housed in a specific pathogen-free facility. Animal experiments were approved by the University of Minnesota Institutional Animal Care and Use Committee.

### Cell preparation and flow cytometry

Immune cells were isolated from the liver, as previously described.^25^ For flow cytometric analysis, cells were incubated with fluorophore-conjugated primary antibodies for 30 minutes at 4 °C, as previously described.^26^ For intracellular staining of transcription factors, cells were stained with antibodies to surface makers, then fixed and permeabilized with True-Nuclear Transcription Factor Buffer Set (Biolegend). Cells were incubated with antibodies to transcription factors. Viability dye LIVE/DEAD Fixable Near IR (780) Viability Kit was from Thermo Fisher Scientific. Biotinylated CD1d-PBS57 monomer was obtained from NIH tetramer core, tetramerized using Streptavidin-PE (Thermo Fisher Scientific), and CD1d-PBS57 tetramer was then used to detect invariant natural killer T (iNKT) cells, as previously described.^27^,^28^ Flow cytometry data were acquired on a BD Fortessa (BD Biosciences) and analyzed using FlowJo software.

### Single-cell RNA sequencing and bioinformatic analysis

Cells from individual samples were tagged with 0.5 μg of TotalSeq-A hashtag antibodies (BioLegend), pooled, and loaded into a 10× Genomics chip. Libraries for gene expression and hashtag oligos were sequenced on a NovaSeq S4 platform (2×150 bp PE), as we previously described.^29^ We used Cell Ranger (10× Genomics) for feature quantification and Seurat for downstream analyses. Uniform manifold approximation and projection were utilized for a visual representation of clusters. Differential expression (DE) between groups was assessed using the standard Wilcoxon rank-sum test. Gene set enrichment analysis (GSEA) was performed using clusterProfiler in R with Log_2_ FC ranked differentially expressed genes. The volcano plot of differentially expressed genes was generated using ggplot2 in R. Cell-to-cell communication inference was performed using the CellChat R package (v1.6.1) to determine the interactions between T cells and B cells in cluster 2. For The Cancer Genome Atlas (TCGA) survival analysis, we modeled the impact of cluster 2 B cell abundance on the overall survival of liver cancer patients (LIHC dataset), following a described methodology.^30^ The scRNA-seq data has been deposited in the Gene Expression Omnibus (GEO), accession number GSE276982.

### Histology

Liver sections fixed in 10% formalin were evaluated for steatosis and fibrosis using fast-green Sirius-Red and hematoxylin and eosin staining, some sections were stained for alpha-fetoprotein (AFP) and GP100, performed by the Biorepository and Laboratory Services, and the Comparative Pathology Shared Resource at the University of Minnesota. The collagen fraction in Sirius-Red-stained images was quantified using the Weka Trainable Segmentation plugin in FIJI software (ImageJ). Red-stained collagen was segmented and isolated using color thresholding. The percentage area positive for collagen was calculated for each section.

### ALT and AST

Serum ALT and AST levels were measured using a clinical chemistry analyzer (Beckman Coulter AU480).

### Intrasplenic injection of B16 tumor cells

Mice were anesthetized, and an incision was made at the left flank to externalize the spleen. 3×10^5^ B16F10 tumor cells suspended in 50 μL of PBS were injected into the spleen, as previously described.^31^ Splenectomy was then performed using an electrocautery device. The incision was closed with a resorbable suture. The liver was collected 2 weeks later for analysis. The B16F10 tumor cell line was kindly provided by Dr David Masopust at the University of Minnesota.

## RESULTS

### MASH-driven HCC development in MUP-uPA mice

**FIGURE 1:**
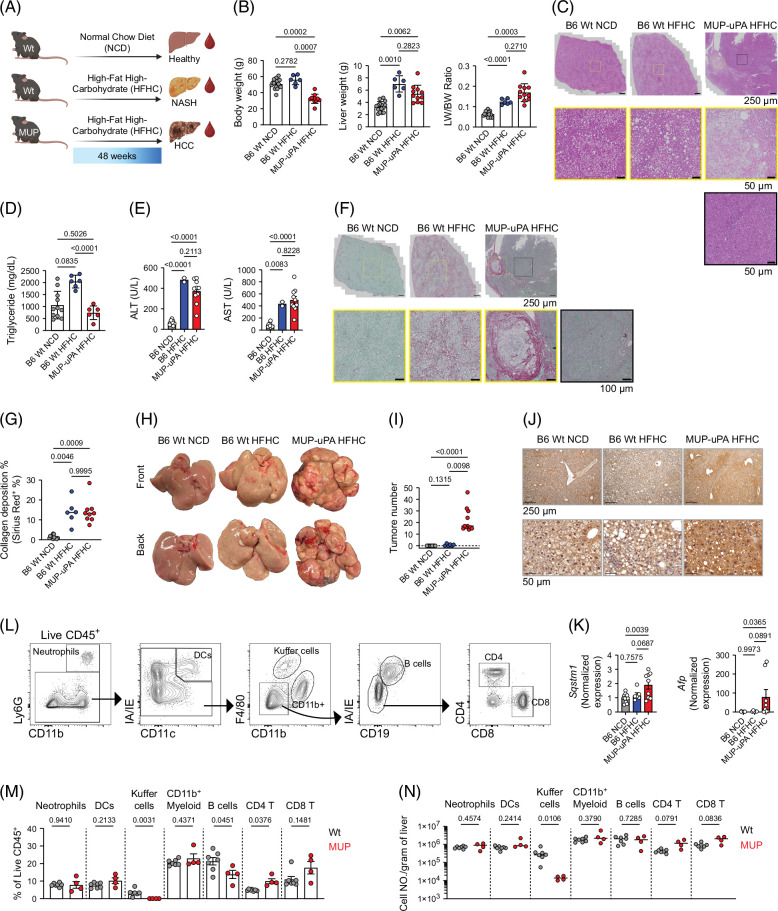
MASH-induced HCC development in MUP-uPA mice. (A) Experimental scheme. (B) Body weight (left), liver weight (middle), and liver-to-body weight ratio (right). (C) Representative hematoxylin and eosin images of livers from B6 Wt normal chow diet (NCD), B6 Wt high-fat high-carbohydrate diet (HFHC), and MUP-uPA HFHC mice. The corresponding magnified images are at the bottom. In the MUP-uPA HFHC image, the yellow square outlined the tumor region, while the black square outlined the non-tumor region. (D) Liver tissue triglyceride level. (E) Serum ALT (left) and AST (right) level. (F) Representative fast-green Sirius-Red images of livers from B6 Wt NCD, B6 Wt HFHC, and MUP-uPA HFHC mice. The corresponding magnified images are at the bottom. In the MUP-uPA HFHC image, the yellow square outlined the tumor region, while the black square outlined the non-tumor region. (G) Quantification of the area with collagen deposition. (H) Representative images of the front and back views of livers collected from B6 Wt NCD, B6 Wt HFHC, and MUP-uPA HFHC mice. (I) Tumor quantification. (J) Representative immunohistochemistry images of AFP staining of livers from B6 Wt NCD, B6 Wt HFHC, and MUP-uPA HFHC mice. The corresponding magnified images are at the bottom. (K) Real-time PCR gene-expression analysis of *Sqstm1* and *Afp* in liver tissues. (L) Representative gating strategy for flow cytometric analysis of liver. (M) Frequency of various immune cell populations in the liver (out of CD45^+^ cells). (N) Cell number of various immune cell populations in the liver (cell number/gram of liver). One-way ANOVA or unpaired *t* test, numbers on top of columns are *p* values. Abbreviations: DCs, dendritic cells; MUP, major urinary protein; uPA, urokinase-type plasminogen activator; Wt, wild type.

To investigate how MASH-driven HCC alters the gene expression profile of intrahepatic immune cells, we fed MUP-uPA mice an HFHC diet for 48 weeks^8^ (Figure 1A). Control groups included C57BL6/J (Wt) mice fed either an NCD or the HFHC diet to induce MASH without HCC (Figure 1A). Compared with Wt controls, MUP-uPA mice gained less body weight in response to the HFHC diet (Figure 1B), likely due to their advanced liver disease. Despite their lower BW, however, MUP-uPA mice exhibited a marked increase in their liver and liver-to-body weight ratios (Figure 1B). MUP-uPA mice also showed limited lipid accumulation, relative to Wt mice, particularly HFHC-fed Wt mice (Figures 1C and D). While the liver from both NCD-fed and HFHC-fed Wt mice exhibited moderate and severe microvesicular steatosis, only the liver of HFHC-fed Wt mice had marked diffuse panlobular macrovesicular steatosis (Figure 1C). Notably, unlike NCD-fed and HFHC-fed Wt mice, the liver from HFHC-fed MUP-uPA mice presented with coalescing lobules of invasive neoplasms composed of large round to polygonal cells. Surrounding the neoplastic lobules, we identified inflammatory cells and infrequent individual cell necrosis (Figure 1C). There was minimal biliary hyperplasia and single-cell hepatocyte necrosis in NCD-fed and HFHC-fed Wt mice (Figure 1C). Serum ALT/AST (Figure 1E) and pericellular fibrosis (Figures 1F and G) were similarly elevated in both MUP-uPA and HFHC-fed Wt, indicative of advanced MASH progression. In addition, MUP-uPA mice exhibited increased fibrosis in tumor-adjacent areas compared to non-tumor regions (Figure 1F), as previously reported.^8^ Notably, the MUP-uPA mice showed evidence of profound HCC development (Figure 1H), including an increased number of tumors (Figure 1I), higher expression of the tumor-associated marker AFP (Figure 1J), and elevated expression of the tumorigenesis-related genes Sqstm1 (p62) and Afp (Figure 1K). In contrast, Wt mice fed either the NCD or HFHC diets exhibited no evidence of HCC development, in agreement with previous reports.^7^,^8^,^23^ We also performed flow cytometry to quantify the relative frequency and abundance of the major immune cell types using an established gating strategy (Figure 1L). We found that MASH-driven HCC livers had a marked loss of Kupffer cells and an increased accumulation of CD4 and CD8 T cells, while neutrophils, DCs, and CD11b^+^ myeloid cells remained unchanged (Figures 1M and N). In addition, we noted a decrease in the frequency of B cells but not in their absolute number (Figures 1M and N). Together, these observations indicate that MUP-uPA mice fed an HFHC diet develop key characteristics of MASH-driven HCC.

### Single-cell expression profile and cell types of hepatic immune cells in HCC

**FIGURE 2:**
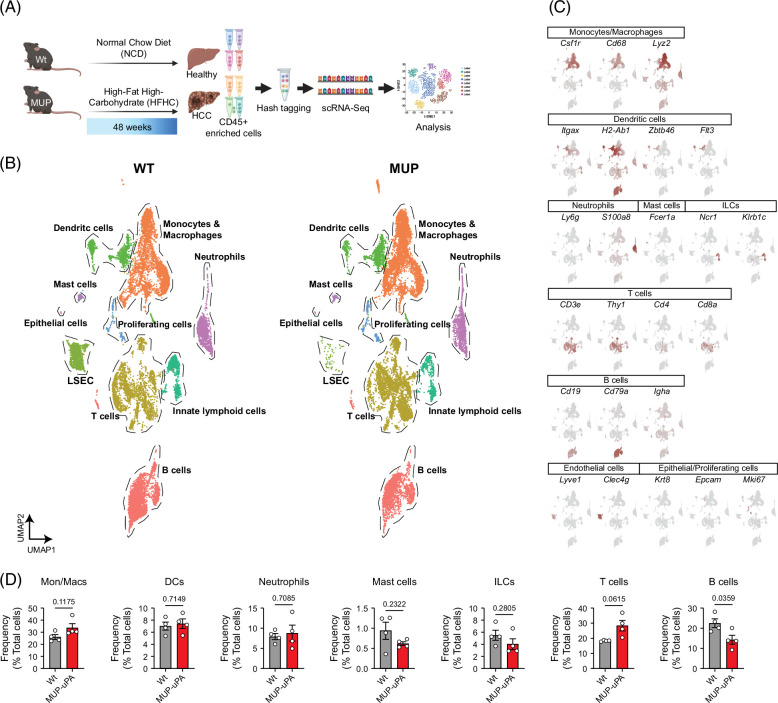
Landscape of immune cell distribution in healthy and HCC livers. (A) Experimental scheme. (B) UMAP visualization of reclustered hepatic immune cells. Twenty-nine clusters were identified that are further grouped into 10 major cell types. (C) Expression of selected marker genes to identify various populations of immune cells. (D) Relative abundance of each population of immune cells in healthy and HCC liver. Unpaired *t* test; numbers on top of columns are *p* values. Abbreviations: DCs, dendritic cells; LSEC, Liver sinusoidal endothelial cell; MUP, major urinary protein; scRNA-seq, single-cell RNA sequencing; uPA, urokinase-type plasminogen activator; UMAP, uniform manifold approximation and projection; WT/Wt, wild type.

To characterize the liver immune cells during MASH-driven HCC, we performed scRNA-seq of CD45^+^ leukocytes purified from the livers of NCD-fed Wt and HFHC-fed MUP-uPA mice (Figure 2A). CD45^+^ cells were barcoded, pooled (n=4 per group), and subjected to droplet-based scRNA-seq, as previously described.^25^ After data processing, we obtained 28,769 cells with unique barcodes, including 14,459 from healthy and 14,310 cells from HCC-affected livers. Unsupervised graph-based clustering with uniform manifold approximation and projection of the cells unveiled 29 distinct clusters (Figure 2B). Using marker gene expression and differentially expressed gene analysis, we classified these cells into major immune subsets, including myeloid cells (*Csf1r*, *Cd68*, and *Lyz2*), DCs (*Itgax*, *H2-Ab1*, *Zbtb46*, and *Flt3*), neutrophils (*Ly6g* and *s100a8*), mast cells (*Fcer1a*), innate lymphoid cells (*Ncr1* and *Krlb1c*), T cells (*Cd4* and *Cd8*), B cells (*Cd19* and *Cd79a*), as well as minor clusters containing contaminating nonimmune cells (Figures 2B and C). Quantification showed an increased frequency of total T cells in the HCC liver, while B cells declined (Figure 2D), suggesting some alterations in the immune cell composition during MASH-driven HCC. Given the heterogeneity of these subsets, we next reclustered and analyzed each major subset independently.

### Functional insights into the B cell subsets in HCC revealed by scRNA-seq

**FIGURE 3:**
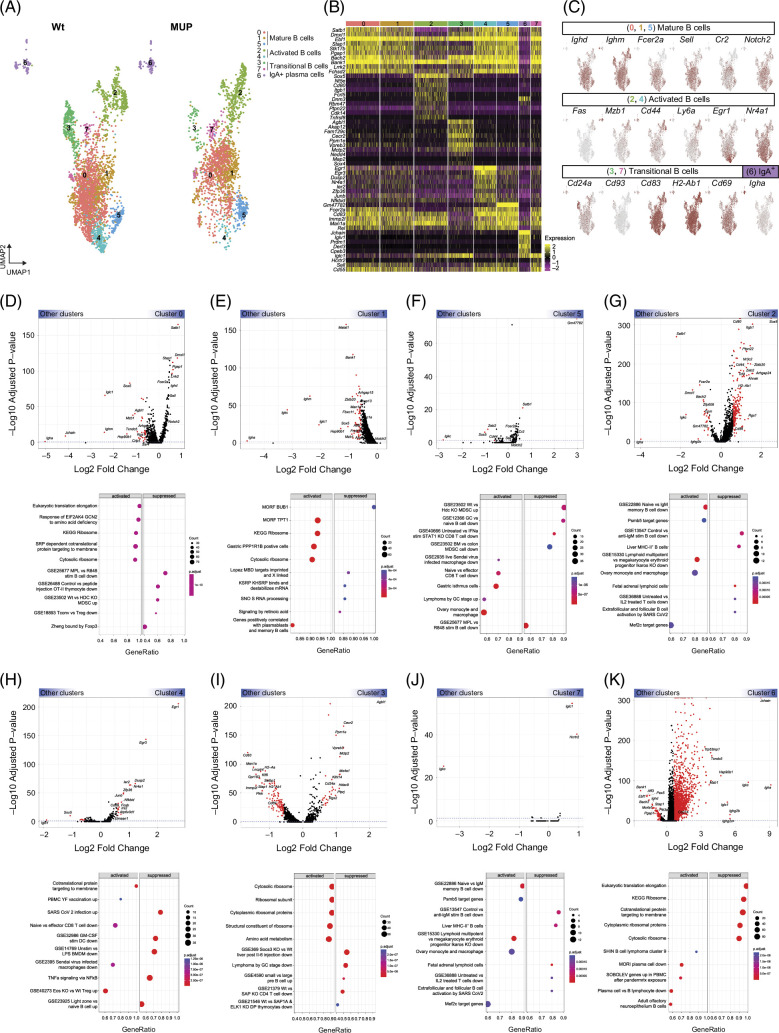
Heterogeneity and pathway enrichment analysis of B cells. (A) UMAP visualization of reclustered monocytes and macrophages population. Eight clusters were identified that are further grouped into 4 major cell types. (B) Heatmap of top 10 highly expressed genes within clusters using unsupervised clustering. (C) Expression of selected marker genes to identify various B cell clusters. (D–K) Volcano plot of gene expression analysis of clusters 0, 1, 5, 2, 4, 3, 7, 6 (top), as well as GSEA of Log_2_ FC ranked DEGs for clusters 0, 1, 5, 2, 4, 3, 7, 6 (bottom) showing the top 5 activated and suppressed pathways. Abbreviations: DEGs, differentially expressed genes; GSEA, gene set enrichment analysis; MUP, major urinary protein; UMAP, uniform manifold approximation and projection; Wt, wild type.

We uncovered 8 clusters with distinct transcriptional patterns (Figures 3A and B) that we manually categorized into 4 subtypes of B cells, namely mature (0, 1, and 5), activated (2 and 4), transitional (3 and 7), and IgA^+^ plasma cells (6), based on marker gene expression (Figure 3C).

#### Mature B cells

Cluster 0 showed high expression of genes and pathways implicated in transcriptional regulation, protein synthesis, signal transduction, and metabolic pathways such as *Satb1*, *Dmxl1*, *Pgap1*, *Stap1*, *Ebf1*, and *Bach2* (Figure 3D, top), suggesting these cells are engaged in regulatory activities and heightened activation in MASH-driven HCC (Figure 3D, bottom). Cluster 1 showed lower expression of genes involved in the immune response, including *Zbtb20*, *Mzb1*, *Sox5*, *Iglc1*, *Ighm*, *Igkc*, *Jchain*, and *Igha* (Figure 3E, top), essential for immunoglobulin production. GSEA revealed decreased cellular activity with a marked suppression in pathways integral to cell cycle regulation, RNA stability and processing, and retinoic acid signaling (Figure 3E, bottom). Similarly, cluster 5 showed lower expression of genes associated with activation, transcriptional regulation, and differentiation, such as *Cd44*, *Crip1*, *Zeb2*, *Sox5*, and *Igkc* (Figure 3F, top). Consistently, GSEA predicted suppression of pathways related to myeloid cell differentiation and immune suppression, B cell maturity, and myeloid-derived suppressor cell activity (Figure 3F, bottom).

#### Activated B cells

Cluster 2 had high expression of genes involved in cell adhesion and co-stimulatory signaling, such as *Sox5*, *Itgb1*, *Zeb2*, *Zbtb20*, *Ptpn22*, *Tcf4*, *CD44*, *CD86*, and *CD80* (Figure 3G, top), suggesting their involvement in interactions with other immune cells. Furthermore, genes associated with B cell identity and function, such as *Bach2*, *Fcer2a*, *Ighg2c*, *Dmxl1*, *Igkc*, *Satb1*, and *Igha*, were downregulated, indicating a shift away from antibody production. Consistently, GSEA predicted activation of protein synthesis pathways and suppression of intracellular transport and B cell memory differentiation pathways (Figure 3G, bottom), suggesting that these cells are oriented toward immediate immune responses rather than long-term memory B cell functions. Cluster 4 upregulated genes involved in growth response, transcriptional regulation, signal transduction, and activation, such as *Egr1*, *Egr3*, *Dusp2*, *Nr4a1*, *Ier2*, *Zfp36*, *Junb*, *Egr2*, *Nfkbid*, and *Cd69* (Figure 3H, top). GSEA predicted heightened protein trafficking, similar to effector T cells involved in the antitumor response, but showed downregulation of inflammatory and B cell differentiation pathways (Figure 3H, bottom), suggesting a suppressive role in inflammatory signaling.

#### Transitional B cells

Cluster 3 was enriched for genes involved in signal transduction, chromatin remodeling, and intracellular trafficking, such as *Ms4a1*, *Mctp2*, *Hdac9*, *Ppm1e*, *Rgs2*, and *Ptprj*, observed in intracellular regulatory processes. Furthermore, these cells had a lower expression of genes related to antigen presentation and lymphocyte signaling, including *H2-Aa*, *Stap1*, *Klf6*, and *Cd83* (Figure 3I, top). GSEA revealed activation of protein synthesis pathways and metabolism, suggesting enhanced protein production and metabolic processing. Furthermore, GSEA indicated suppression of pathways related to cytokine signaling and lymphocyte function (Figure 3I, bottom). In contrast, cluster 7 showed minimal transcriptional changes compared to the general B cell population, suggesting a relatively stable expression profile due to their intermediary role in B cell maturation (Figure 3J, top, bottom).

#### IgA^+^ B cells

Cluster 6 showed an enrichment of the immunoglobulin genes *Igha*, *Jchain*, *Igkc*, *Ighm*, *Ighg2b*, *Ighg2c*, and *Iglc1*. Notably, these cells upregulated *Mzb1*, which is implicated in plasma cell differentiation and antibody production (Figure 3K, top). The B cells in this cluster also downregulated genes involved in cell activation and signaling (*Pik3ap1*, *Stap1*, *Pgap1*) and B cell development and antigen recognition (*Ms4a1* and *Ighd*). Furthermore, their reduced expression of the key transcription factors *Pax5*, *Bach2*, *Aff3*, *Ebf1*, and *Bank1* indicate differentiation into antibody-secreting plasma cells (Figure 3K, top). Indeed, GSEA showed suppressed pathways involved in translation and protein targeting, indicating a specialization in antibody secretion over cellular proliferation or antigen presentation (Figure 3K, bottom). Overall, our scRNA-seq analysis revealed gene expression patterns typical of mature, activated, transitional, and plasma B-cell subpopulations during MASH-driven HCC.

### Essential role of B cells in the resistance against MASH-driven HCC

**FIGURE 4:**
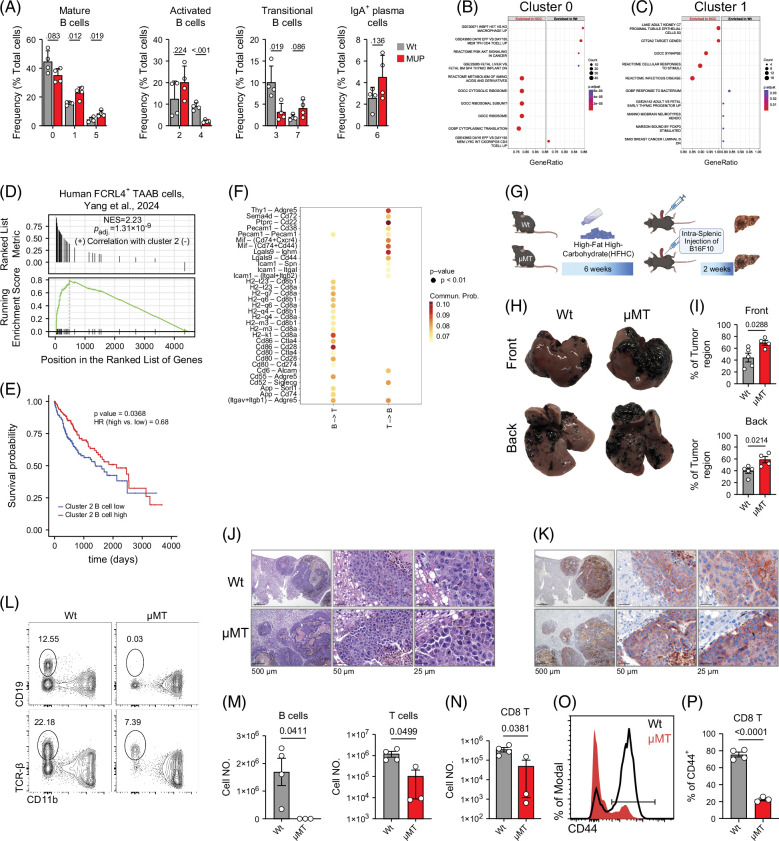
B cells ameliorate hepatic tumor progression. (A) Relative abundance of each B cell cluster in healthy and HCC liver. (B) GSEA of Log_2_ FC ranked DEGs of cluster 0 over other clusters combined in B cells, showing the top 5 activated and suppressed pathways in cluster 0. (C) GSEA of Log_2_ FC ranked DEGs of cluster 2 over other clusters combined in B cells, showing the top 5 activated and suppressed pathways in cluster 2. (D) GSEA enrichment plot of cluster 2 B cells to human FCRL4^+^ TAAB cells. (E) TCGA survival analysis of the overall survival of liver cancer patients (LIHC dataset) stratified by cluster 2 B cell abundance. (F) Significant ligand–receptor pairs between cluster 2 B cells and T cells within HCC. (G) Experimental scheme. (H) Representative pictures of the front (top) and back (bottom) views of livers collected from Wt and μMT mice. (I) Quantification of tumor region on the surface of the liver on the front (top) and back (bottom). (J) Representative H&E images of livers from Wt (top) and μMT (bottom) mice. The corresponding magnified images are in the middle and right. (K) Representative immunohistochemistry images of GP100 staining of livers from Wt (top) and μMT (bottom) mice. The corresponding magnified images are in the middle and right. (L) Representative flow cytometric plots for B cell and T cell gating. (M, N) Cell number of total B cells (M, right), T cells (M, left), and CD8^+^ T cells (N) in healthy and HCC liver. (O, P) Flow cytometric histogram of CD44 expression in CD8 T cells from Wt and μMT mice (O); frequency of CD44^+^ cells among CD8^+^ T cells (P). Abbreviations: DEGs, differentially expressed genes; GSEA, gene set enrichment analysis; H&E, hematoxylin and eosin; MUP, major urinary protein; TAAB, tumor-associated atypical B; TCGA, The Cancer Genome Atlas; UMAP, uniform manifold approximation and projection; Wt, wild type.

We next determined how MASH-driven HCC alters the abundance and gene expression profile of B cell clusters. In MUP-uPA mice, the frequency of mature B cells with regulatory activity (0) decreased, while that of B cells with reduced activity (1 and 5) increased (Figure 4A), indicating a suppression of B cell function. Among activated clusters, B cells that support immediate immune responses (2) increased, while those with a suppressive signature (4) decreased in abundance. Transitional B cells in cluster 3 decreased while those in cluster 7 expanded in MUP-uPA mice, suggesting diminished B cell activation. Finally, there was a trend for increased IgA^+^ plasma cells in the HCC livers, in agreement with a previous report^8^ (Figure 4A). We next performed DE analysis between Wt and MUP-uPA for each B cell cluster. We found substantial differences in gene expression in clusters 0, 1 (mature B cells), and 2 (activated B cells), while clusters 3, 4, 5, 6, and 7 were not altered between groups (Supplemental Figure S1A, http://links.lww.com/HC9/B931). GSEA showed the mature B cells in cluster 0 from MUP-uPA mice had enriched pathways related to cellular metabolic processes and protein synthesis machinery (Figure 4B), suggesting heightened cellular activity reflective of a robust antigen-processing and presentation capability. Similarly, mature B cells in cluster 1 displayed enriched pathways linked to protein translation and elongation in MUP-uPA mice, compared with Wt controls (Figure 4C). Considering that activated B cells in cluster 2 resembled tumor-associated atypical B (TAAB) cells, a subset of B cells that stimulate other immune cells in human HCC^30^ (Figure 3G), we further assessed this population. GSEA analysis using the published TAAB gene signature in human HCC B cells^30^ showed that the gene expression profile of B cells in cluster 2 is highly similar to that of human TAAB cells (Figure 4D). Next, we examined the association between the cluster 2 B cell gene program and the overall survival of liver cancer patients (LIHC) in the Cancer Genome Atlas dataset. After correcting for the effect of cell abundance, we found that a higher expression of the cluster 2 B cell signature correlated with improved survival in patients with liver cancer (Figure 4E). In addition, CellChat intercellular interaction analysis showed that the B cells in cluster 2 interact strongly with CD8^+^ T cells (Figure 4F), in agreement with the notion that TAAB cells support antitumor T cell functions.^30^

To directly investigate the role of B cells in antitumor immunity in the liver, we utilized B cell-deficient μMT mice and a murine model of liver cancer induced by intrasplenic injection of B16F10 melanoma cells. Six-week-old μMT mice and age-matched Wt controls were subjected to an HFHC diet for 6 weeks before tumor inoculation (Figure 4G). Compared with Wt controls, μMT mice showed increased liver metastasis (Figures 4H and I). Histological analysis confirmed the metastasis of B16 melanoma to the liver of Wt and μMT mice, evidenced by a multilobulated invasive neoplasm with regional necrosis (Figure 4J) and staining for GP100 (Figure 4K). Furthermore, μMT mice showed a substantial reduction in T cells, particularly CD8^+^ T cells (Figures 4L–N). The intrahepatic CD8^+^ T cells in μMT mice had lower CD44 expression, suggesting an impaired activation (Figures 4O and P). Overall, the pronounced metastatic tumor burden in B cell-deficient μMT mice showed that B cells are required to control tumor progression in the liver, likely through enhancing recruitment and activation of intrahepatic T cells.

### Transcriptional profiling and functional divergence of T cell subsets in HCC

**FIGURE 5:**
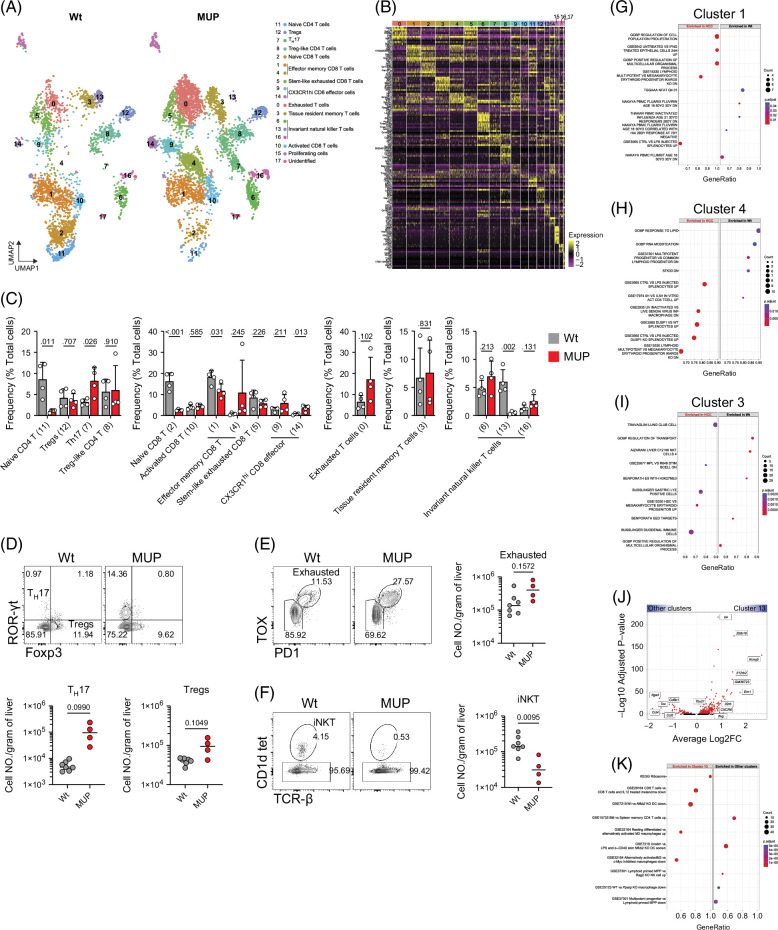
Heterogeneity and pathway enrichment analysis of T cells. (A) UMAP visualization of reclustered T cells population. Eighteen clusters were identified that are further grouped into 13 major cell types. (B) Heatmap of top 10 highly expressed genes within clusters using unsupervised clustering. (C) Relative abundance of each cluster of cells in healthy and HCC liver. (D) Flow cytometric analysis of T_H_17 and Tregs cells in total CD4 T cells (top); cell number of T_H_17 (bottom left) and Tregs (bottom right) cells per gram of liver tissue. (E) Flow cytometric analysis of exhausted CD8 T cells in total CD8 T cells (left and middle); cell number of exhausted CD8 T cells per gram of liver tissue (right). (F) Flow cytometric analysis of iNKT cells (left and middle); cell number of exhausted iNKT cells per gram of liver tissue (right). (G–I) GSEA of Log_2_ FC ranked DEGs of HCC over healthy, showing the top 5 activated and suppressed pathways in clusters 1 (G), 4 (H), and 3 (I). (J) Volcano plot of gene expression analysis of cluster 13 over all other clusters. (K) GSEA of Log_2_ FC ranked DEGs of cluster 13 over all other clusters showing the top 5 activated and suppressed pathways. Unpaired *t* test, numbers on top of columns are *p* values. Abbreviations: DEGs, differentially expressed genes; GSEA, gene set enrichment analysis; iNKT, invariant natural killer T; MUP, major urinary protein; UMAP, uniform manifold approximation and projection; Wt, wild type.

We performed unsupervised uniform manifold approximation and projection analysis of T cells and identified 18 unique clusters (Figure 5A), stratified into 13 primary T cell subsets based on marker gene expression and DE analysis. These subsets included naïve CD4^+^ T cells (11), regulatory CD4^+^ T cells (Tregs) (12), T helper 17 (T_H_17) cells (7), Treg-like CD4^+^ T cells (8), naïve CD8^+^ T cells (2), effector memory CD8^+^ T cells (1 and 4), stem-like exhausted CD8^+^ T cells (5), CX3CR1^hi^ CD8^+^ effector cells (9 and 14), exhausted T cells (0), tissue-resident memory T cells (cluster 3), iNKT cells (6, 13, and 16), activated CD8^+^ T cells (10), proliferating cells (15), and 1 cluster (17) identified as contaminating non-T cells (Figures 5A and B and Supplemental Figure S2B, http://links.lww.com/HC9/B931). We found a substantial increase in T_H_17 cells and CX3CR1^hi^ CD8^+^ effector cells in HCC livers, suggesting increased inflammation, along with a trending increase in exhausted T cells, indicating immune dysfunction (Figure 5C). Conversely, there was a substantial decline in naïve T cells and iNKT cells in HCC livers, suggesting a shift from immunosurveillance toward active but exhausted T cell responses (Figure 5C). There was a decrease in effector memory CD8^+^ T cells (cluster 1), while all other subsets largely remained unchanged (Figure 5C). Flow cytometry analysis confirmed the expansion of T_H_17 and exhausted CD8^+^ T cells and the decline of iNKT cells in HCC livers (Figures 5D–F). DE analysis showed that the most substantial changes in gene expression between healthy and HCC livers occur in effector memory CD8^+^ (clusters 1 and 4) and tissue-resident (cluster 3) T cells (Supplemental Figure S2A, http://links.lww.com/HC9/B931). While effector memory CD8^+^ T cells from healthy livers display enrichment in pathways associated with baseline cellular processes, their counterparts from HCC showed enrichment in proliferative and stress response pathways (Figures 5G and H), indicating an active antitumor response with varied states of activation or exhaustion. Tissue-resident memory CD8^+^ T cells in HCC livers exhibit enrichment in pathways indicative of localization within specific tissue microenvironments (Figure 5I), suggesting a site-specific immune response to HCC, while cells from healthy livers are enriched with regulatory and transport pathways, resembling a more traditional role in tissue surveillance (Figure 5I). iNKT cells in cluster 13 have increased expression of genes associated with T_H_1-like functions (*Zbtb16*, *Tbx21*, and *Ifng)* and decreased expression of *Tox*, *Ccl4*, and *Ccl5*, suggesting enhanced cytotoxic and cytokine responses (Figure 5J). These iNKT cells are enriched in cellular biosynthesis and immune response pathways (Figure 5K), suggesting an enhanced activation. We detected minimal changes in gene expression for other T cell clusters, suggesting relative transcriptional stability in these subsets. (Supplemental Figure S2A, http://links.lww.com/HC9/B931). Collectively, these findings underscore a profound remodeling of the T cell compartment in HCC, where the balance of surveillance, activation, and exhaustion reflects the capacity for an effective antitumor response.

### Subtype-specific functional remodeling of myeloid cells in MASH-driven HCC

**FIGURE 6:**
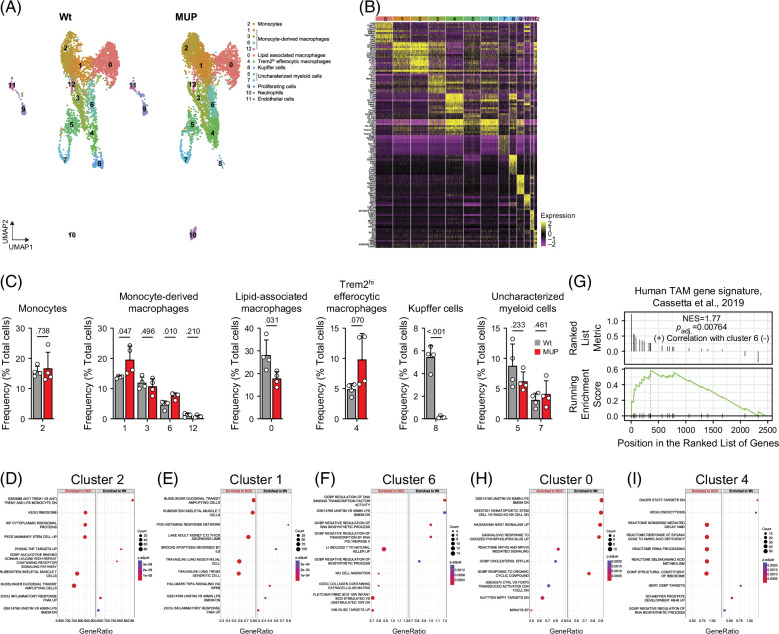
Heterogeneity and pathway enrichment analysis of monocyte and macrophage populations. (A) UMAP visualization of reclustered monocytes and macrophages population. Thirteen clusters were identified that are further grouped into 9 major cell types. (B) Heatmap of top 10 highly expressed genes within clusters using unsupervised clustering. (C) Relative abundance of each cluster of cells in healthy and HCC liver. (D–F) GSEA of Log_2_ FC ranked DEGs of HCC over healthy, showing the top 5 activated and suppressed pathways in clusters 2 (D), 1 (E), and 6 (F). (G) GSEA enrichment plot of cluster 6 monocyte-derived macrophages to human TAMs. (H, I) GSEA of Log_2_ FC ranked DEGs of HCC over healthy, showing the top 5 activated and suppressed pathways in clusters 0 (H) and 4 (I). Unpaired *t* test, numbers on top of columns are *p* values. Abbreviations: DEGs, differentially expressed genes; GSEA, gene set enrichment analysis; MUP, major urinary protein; TAMs, tumor-associated macrophages; UMAP, uniform manifold approximation and projection; Wt, wild type.

The reclustering of myeloid cells revealed 11 clusters, further stratified into 9 subtypes, including monocytes (2), monocyte-derived macrophages (1, 3, 6, and 12), lipid-associated macrophages (0), Trem2^hi^ efferocytic macrophages (4), Kupffer cells (8), unidentifiable myeloid cells (5 and 7), proliferating cells (9), and contaminating neutrophils (10) and endothelial cells (Figures 6A and B and Supplemental Figure S3B, http://links.lww.com/HC9/B931). Quantification of cluster abundance showed that monocytes remained unchanged, and monocyte-derived macrophage (clusters 1 and 6) increased (Figure 6C). While lipid-associated macrophages declined, Trem2^hi^ efferocytic macrophages trended to increase in HCC, suggesting a transition between lipid handling and efferocytosis functions. Consistent with our flow cytometric analysis (Figures 2D and E), Kupffer cells were substantially reduced in HCC livers (Figure 6C). DE and GSEA analysis revealed substantial transcriptomic alterations in clusters 0, 1, 2, 3, 4, 6, and 8 (Supplemental Figure S3A, http://links.lww.com/HC9/B931). Monocytes (cluster 2) showed enrichment in protein synthesis and cellular assembly pathways, indicating an enhanced activation (Figure 6D). Monocyte-derived macrophages (clusters 1 and 6) shifted toward pathways involved in tissue remodeling and immune regulation, characteristics of TAMs^32^,^33^ (Figures 6E and F). Notably, cluster 6 of monocyte-derived macrophages exhibited gene expression profiles similar to human TAMs^33^ (Figure 6G). Lipid-associated macrophages (cluster 0) showed suppression of lipid processing pathways, suggesting a metabolic reprogramming of these cells in HCC (Figure 6H). Trem2^hi^ efferocytic macrophages (cluster 4) displayed enrichment in pathways associated with amino acid metabolism and ribosome function, indicative of a heightened efferocytosis and antigen presentation (Figure 6I). DE analysis revealed minimal changes within clusters 5, 7, and 12, suggesting relative transcriptional stability in these clusters (Supplemental Figure S3A, http://links.lww.com/HC9/B931). Overall, these findings suggest a profound reprogramming of monocytes and macrophage functions in HCC, with cells shifting toward a modulatory role in antitumor immune response.

### Innate lymphoid cell, neutrophil, and dendritic cell dynamics in MASH-driven HCC

Based on their expression of marker genes, we reclustered and classified innate lymphoid cells into conventional NK cells and innate lymphoid cell type 1 (ILC1) (Supplemental Figure S4A–C, http://links.lww.com/HC9/B931). Quantification revealed a decrease in conventional NK cells while ILC1 cells increased (Supplemental Figure S4D, http://links.lww.com/HC9/B931). DE and GSEA analyses (Supplemental Figure S4E, http://links.lww.com/HC9/B931) showed that ILC1 cells were enriched in pathways involved in neural activities and development in HCC, while cells from healthy livers exhibited enrichment in RNA-binding functions (Supplemental Figure S4F, http://links.lww.com/HC9/B931). This suggests a potential shift in ILC1 function from basic cellular processes in HCC.

Reclustering of neutrophils revealed 3 clusters that were classified as inflammatory (0), immune-modulatory (1), and antimicrobial (2) neutrophils, based on marker gene expression and DE analysis (Supplemental Figures 5A–C, http://links.lww.com/HC9/B931). Quantification revealed that antimicrobial neutrophils increased, while both inflammatory and immune-modulatory neutrophils were unchanged (Supplemental Figure S5D, http://links.lww.com/HC9/B931). DE analysis showed substantial changes in gene expression in inflammatory neutrophils (cluster 0) (Supplemental Figure S5E, http://links.lww.com/HC9/B931). GSEA revealed that inflammatory neutrophils had heightened activation pathways in HCC, whereas tissue development and cell adhesion pathways were enriched in healthy livers (Supplemental Figure S5F, http://links.lww.com/HC9/B931). Notably, antimicrobial neutrophils showed enriched pathways related to cellular protein machinery while showing reduced involvement in anti-inflammatory pathways (Supplemental Figure S5G, http://links.lww.com/HC9/B931). These observations suggest that neutrophil populations undergo a functional shift in MASH-driven HCC.

We identified 7 clusters of DCs with unique gene expression profiles that we categorized into 4 main DC subtypes, namely classical dendritic cells type 2 (cDC2), classical dendritic cells type 1 (cDC1), activated DCs, and plasmacytoid dendritic cells (pDCs) (Supplemental Figures S6A–C, http://links.lww.com/HC9/B931). We found a reduction in cDC1 and pDC subsets, whereas the activated DCs remained unchanged and cDC2 increased (Supplemental Figure S6D, http://links.lww.com/HC9/B931). DE analysis showed that clusters 0, 1, and 2 had moderate transcriptional reprogramming (Supplemental Figure S6E, http://links.lww.com/HC9/B931). GSEA showed that cDC2s (0 and 1) had an upregulation in pathways linked to broad immune and developmental processes in HCC, while pathways governing immune system activation were enriched in healthy livers (Supplemental Figures S6F and G, http://links.lww.com/HC9/B931). Furthermore, cDC1s (2) had upregulated proteolytic pathways in HCC, indicative of a potential increase in antigen-processing activities, in contrast with the healthy state characterized by enriched pathways related to cellular stress responses and immune regulation (Supplemental Figure 6H, http://links.lww.com/HC9/B931). These findings revealed a complex reconfiguration of DCs in HCC, possibly contributing to altered immune reactivity and tumor progression.

## DISCUSSION

Although B cells are traditionally known for their essential role in humoral immunity, they are now recognized for their multifaceted functions in liver diseases such as MASH and HCC.^15^,^25^,^34^,^35^ In this study, we found that B cells display a remarkable heterogeneity during MASH-driven HCC. For example, we identified a substantial activation of several B cell subsets, potentially reflecting a pathological role in the development of HCC, as we previously have shown in MASH.^25^ Specifically, we observed an expansion of activated B cells enriched in co-stimulatory and antigen presentation genes in HCC. Indeed, preclinical studies have shown that certain B cell subsets, especially IgA^+^ cells, may disrupt anticancer immunity.^8^ In agreement with this finding, high infiltration of IgG^+^ and IgA^+^ plasma cells correlates with poor HCC prognosis.^34^ However, we also show that B cells express gene programs suggestive of key roles in resisting tumorigenesis. In general, B cells exert their antitumoral functions through antibody production, antigen presentation, and orchestration of T cell responses.^15^,^35^ Clinically, a high density of tumor-infiltrating B cells and B cell-rich tertiary lymphoid structures correlates with a better prognosis of HCC.^36^,^37^,^38^ Our finding that B cell deficiency aggravates MASH-driven HCC suggests that B cell antitumor functions overweight their pro-tumor counterparts and highlight specific B cell subsets as potential targets for immunomodulatory therapies in HCC.

In the current study, we found a decreased accumulation of intrahepatic lymphocytes and substantially impaired activation of CD8^+^ T cells in μMT mice, suggesting that intrahepatic B cells promote antitumor immunity by enhancing T cell activation. This aligns with previous findings that B cells boost antitumor T cell responses by presenting tumor antigens and providing co-stimulatory signals.^35^,^39^,^40^,^41^ B cells can also produce antibodies that promote antigen presentation or directly kill tumor cells^35^,^39^,^40^ and secrete cytokines and chemokines that recruit and activate other immune cells to enhance antitumor response.^39^,^41^ However, certain B cell subsets produce immunosuppressive cytokines such as IL-10 and TGF-β that inhibit T cell activity.^39^ Moreover, immune complexes formed by antibodies can promote inflammation and angiogenesis, facilitating tumor growth and metastasis.^42^ This dual role of B cells highlights their complexity within the tumor microenvironment. While B cell subsets involved in antigen presentation and co-stimulatory signaling can enhance antitumor immunity, other subsets, such as IgA^+^ plasma cells, suppress it. The overall protective role of B cells observed in our study may result from a predominance of anti-tumorigenic subsets or their effective orchestration of immune responses against HCC.

While the MUP-uPA mouse model mimics several key features of human HCC,^7^,^8^,^23^ it presents several limitations. Although the increased hepatocyte endoplasmic reticulum stress and liver damage caused by excessive uPA expression in MUP-uPA mice are features of MASH-driven HCC,^7^,^43^ the MUP-uPA genotype itself may alter the immune cell landscape of the HCC liver. In addition, the MUP-uPA might not capture all possible phenotypes observed in human HCC. Mutations commonly found in human HCC, such as TP53 or CTNNB1,^44^ may exhibit distinct immune landscapes due to their unique oncogenic pathways. TP53 mutations are associated with genomic instability and can alter antigen presentation,^45^ CTNNB1 mutations can affect Wnt/β-catenin signaling pathways known to modulate immune responses.^44^ Lastly, the MUP-uPA mouse model requires a prolonged dietary treatment for HCC development. Therefore, while the MUP-uPA mouse model combined with a Western diet faithfully mimics the human condition and can be used to investigate MASH-driven HCC, caution is warranted when extrapolating these results to human HCC.

Another limitation of our study is that we lack information about the precise localization of the immune cells in the liver. Given that we characterized the immune cells from the whole liver, we are not able to differentiate between liver resident and tumor-infiltrating lymphocytes. Immune cells may localize in tumor-adjacent or non-tumor regions. Thus, we refer to the immune cells in our analysis as intrahepatic immune cells, rather than tumor-infiltrating cells. Future studies employing spatial profiling, such as spatial transcriptomics, are needed to delineate the specific contributions of tumor-infiltrating versus tumor-adjacent immune cells in MASH-driven HCC. Finally, the mechanisms underpinning the protective role of B cells remain to be investigated. Considering the complexity of the immune response during HCC, B cell-mediated regulation of cancer likely involves interactions with other immune cells. Such interactions require further studies to fully understand the contribution of B cells to HCC.

Overall, our study characterizes the immune landscape of MASH-driven HCC as a dynamic and interactive environment, where B cells play a critical protective role against tumorigenesis. In addition, this work provides a resource to study the population dynamics and transcriptional reprogramming of immune cells in MASH-driven HCC.

## Supplementary Material
