# Supplementary material for: Single-cell RNA sequencing reveals a reprogramming of hepatic immune cells and a protective role for B cells in MASH-driven HCC
Source: Hepatol Commun. 2025 Apr 21;9(5):e0668. doi: 10.1097/HC9.0000000000000668 (PMC12014033; doi:10.1097/HC9.0000000000000668)

**Supplementary Figure 1 DEGs analysis of B cell clusters**

(**A**) Volcano plot of gene expression analysis of B cell clusters 0-7 between cells from healthy and HCC livers.


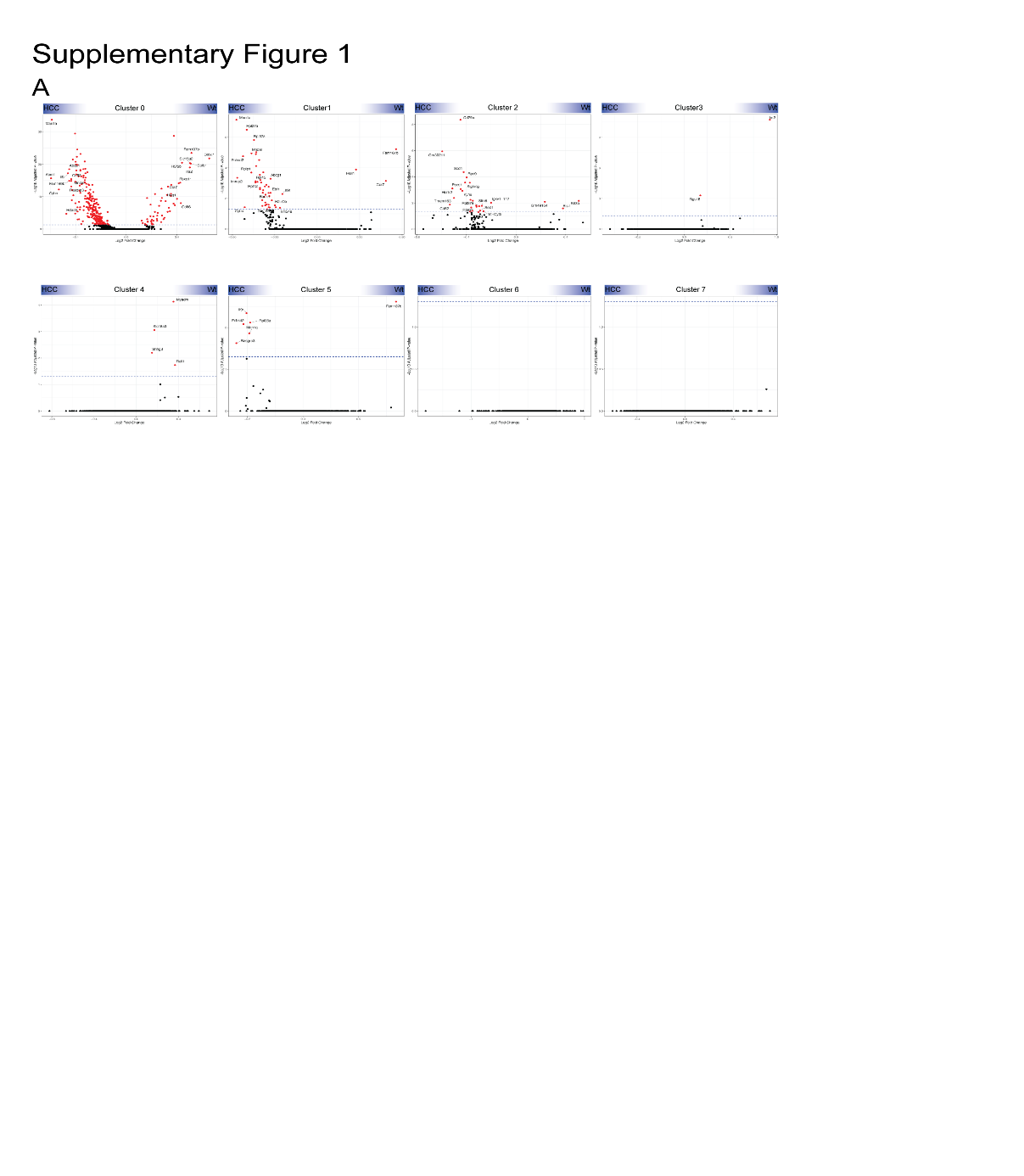


**Supplementary Figure 2 DEGs analysis of T cell clusters**

(**A**) Volcano plot of gene expression analysis of T cell clusters 0-14 and 16 between cells from healthy and HCC livers. (**B**) Expression of selected marker genes to identify various T cell clusters.


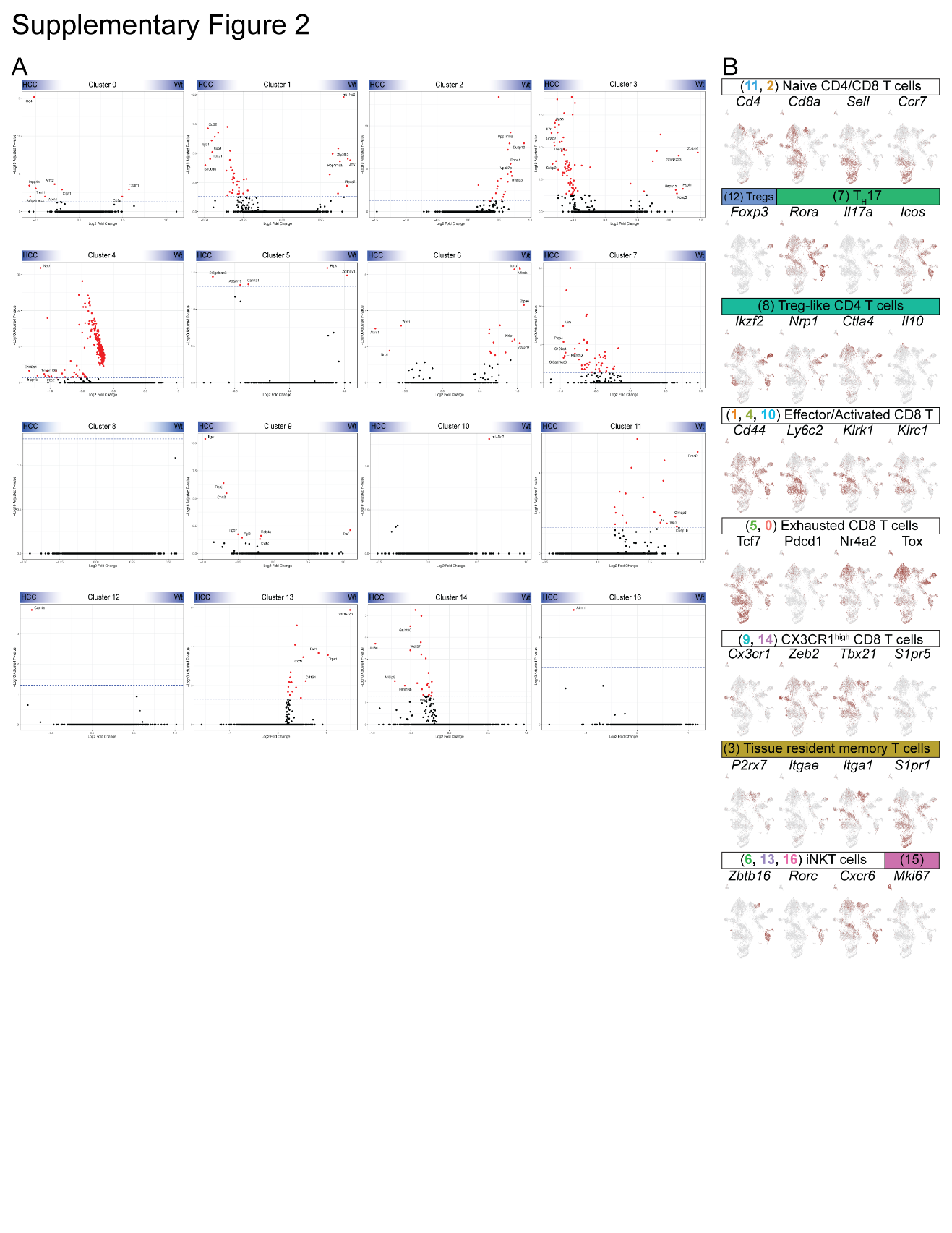


**Supplementary Figure 3 DEGs analysis of Monocytes/macrophage clusters**

(**A**) Volcano plot of gene expression analysis of Monocytes/macrophage clusters 0-8 and 12 between cells from healthy and HCC livers. (**B**) Expression of selected marker genes to identify various myeloid cell clusters.


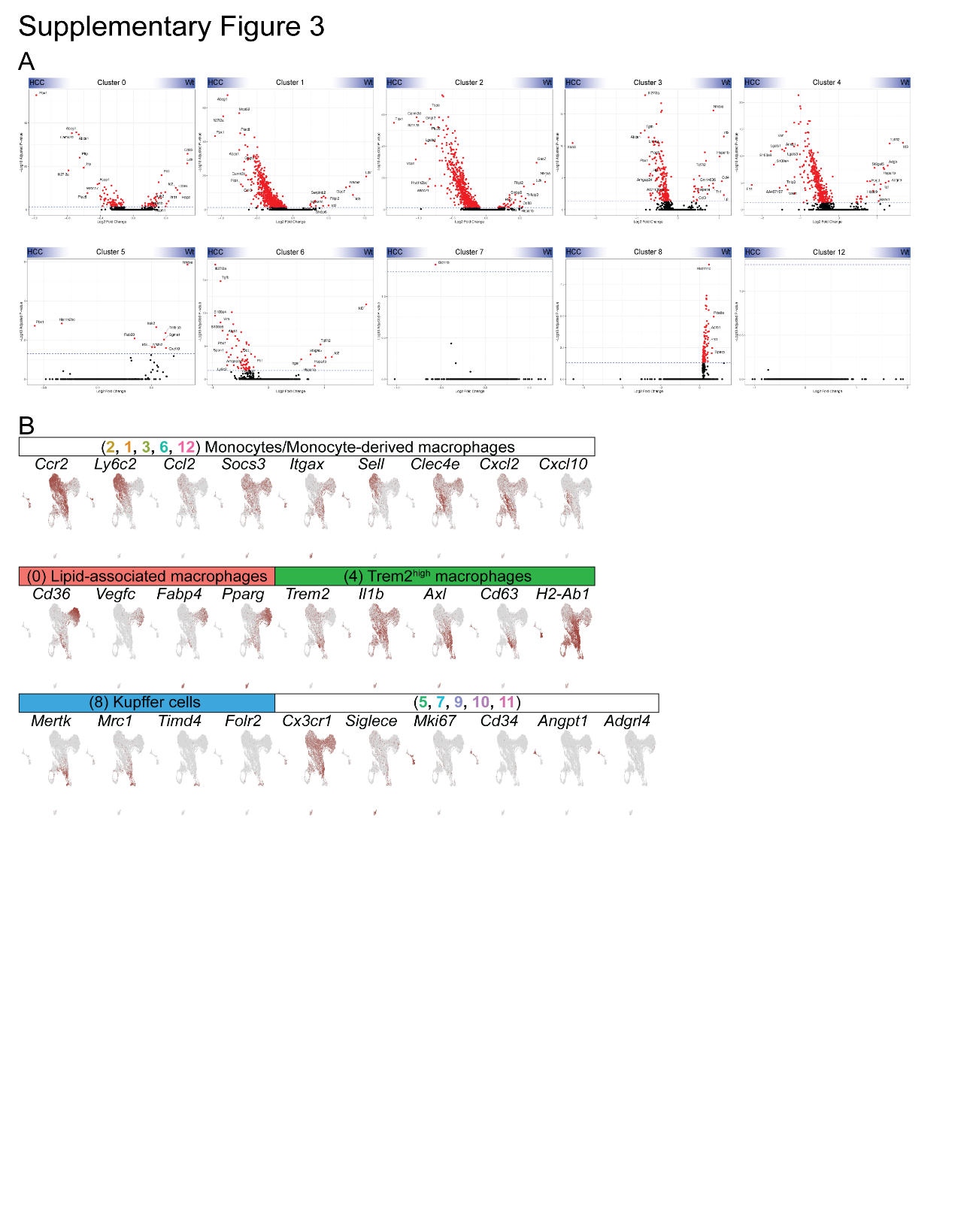


**Supplementary Figure 4 Heterogeneity and Pathway Enrichment Analysis of Innate Lymphoid Cells Populations**

1. Uniform manifold approximation and projection (UMAP) visualization of reclustered innate lymphoid cell (ILC) population. Two clusters were identified. (**B**) Heatmap of top 10 highly expressed genes within clusters using unsupervised clustering. (**C**) Expression of selected marker genes to identify various ILC clusters. (**D**) Relative abundance of each cluster of cells in healthy and HCC liver. (**E**) Volcano plot of gene expression analysis of ILC clusters 0 and 1 between cells from healthy and HCC livers. (**F**) GSEA of Log_2_FC ranked DEGs of HCC over healthy, showing the top 5 activated and suppressed pathways in Cluster 1. Unpaired *t* test, numbers on top of columns are P values.


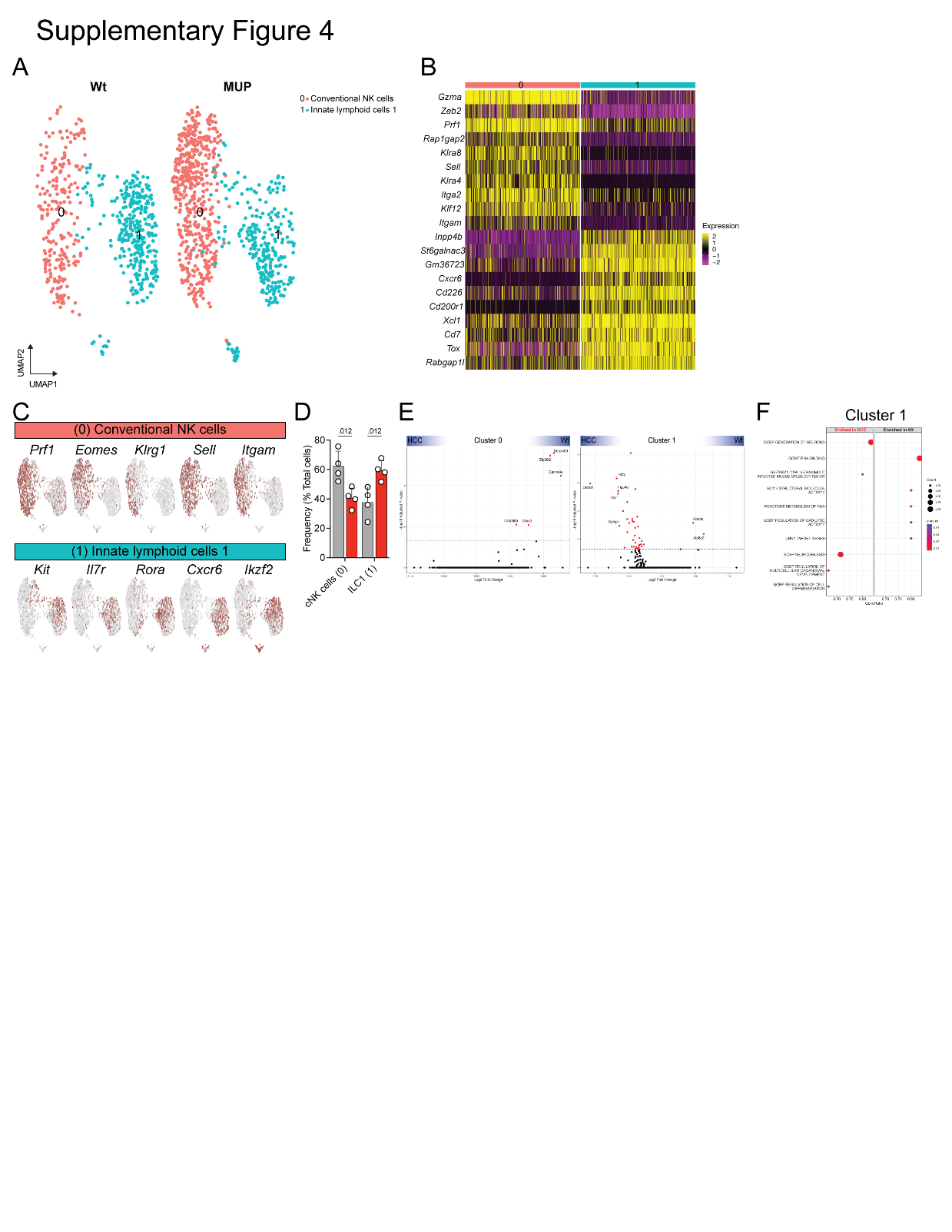


**Supplementary Figure 5 Heterogeneity and Pathway Enrichment Analysis of Neutrophils**

(**A**) Uniform manifold approximation and projection (UMAP) visualization of reclustered neutrophils population. Three clusters were identified. (**B**) Heatmap of top 10 highly expressed genes within clusters using unsupervised clustering. (**C**) Expression of selected marker genes to identify various neutrophil clusters. (**D**) Relative abundance of each cluster of cells in healthy and HCC liver. (**E**) Volcano plot of gene expression analysis of neutrophil clusters 0-2 between cells from healthy and HCC livers. (**F**) GSEA of Log_2_FC ranked DEGs of HCC over healthy, showing the top 5 activated and suppressed pathways in Cluster 0. (**G**) GSEA of Log_2_FC ranked DEGs of Cluster 2 over other Clusters combined, showing the top 5 activated and suppressed pathways in Cluster 2. Unpaired *t* test, numbers on top of columns are P values.


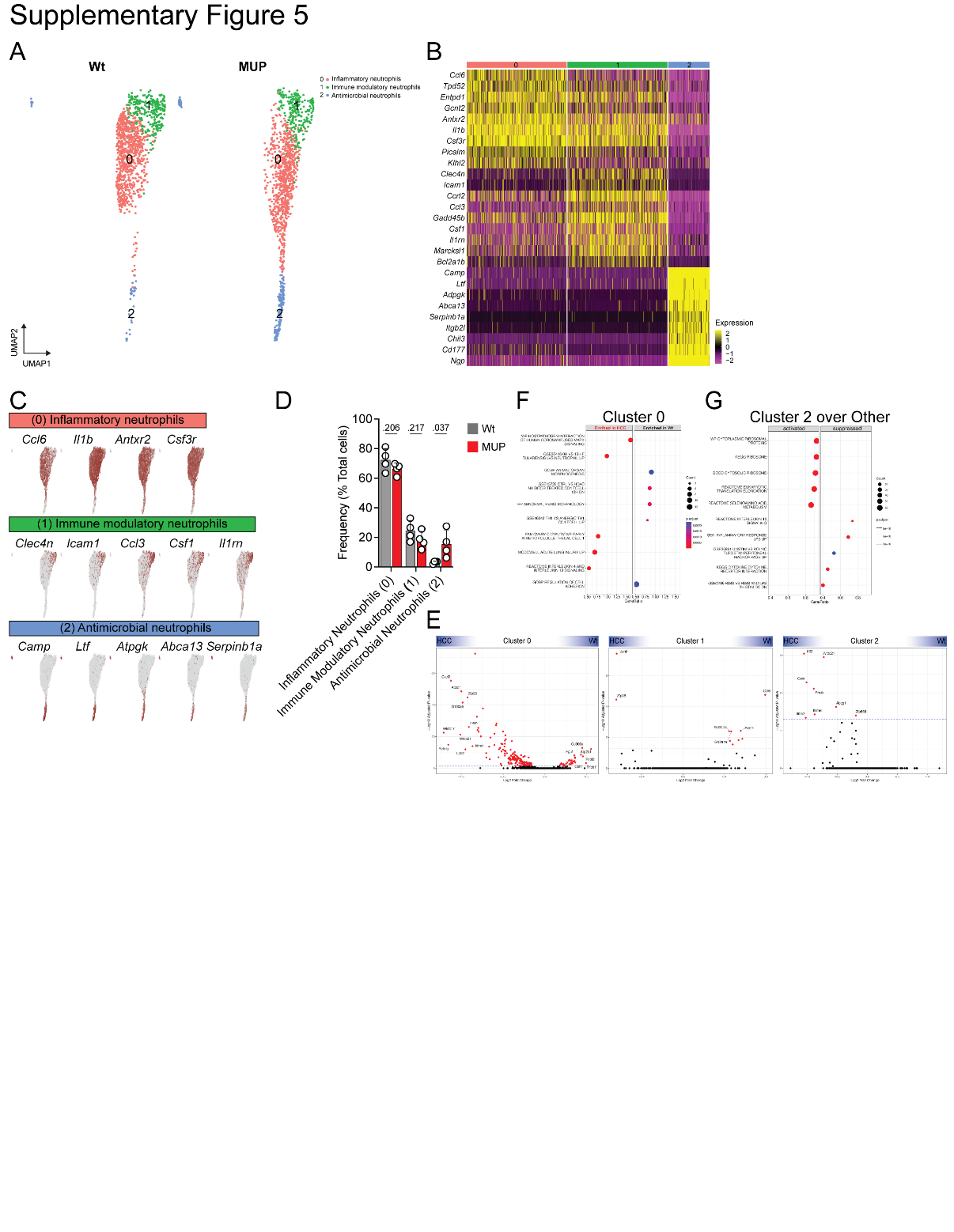


**Supplementary Figure 6 Heterogeneity and Pathway Enrichment Analysis of DCs Populations**

(**A**) Uniform manifold approximation and projection (UMAP) visualization of reclustered DCs population. Seven clusters were identified that are further grouped into four major cell types. (**B**) Heatmap of top 10 highly expressed genes within clusters using unsupervised clustering. (**C**) Expression of selected marker genes to identify various DC clusters. (**D**) Relative abundance of each cluster of cells in healthy and HCC liver. (**E**) Volcano plot of gene expression analysis of neutrophil clusters 0-6 between cells from healthy and HCC livers. (**F-H**) GSEA of Log_2_FC ranked DEGs of HCC over healthy, showing the top 5 activated and suppressed pathways in Cluster 0 (F), 1 (G), 2 (H). Unpaired *t* test, numbers on top of columns are P values.


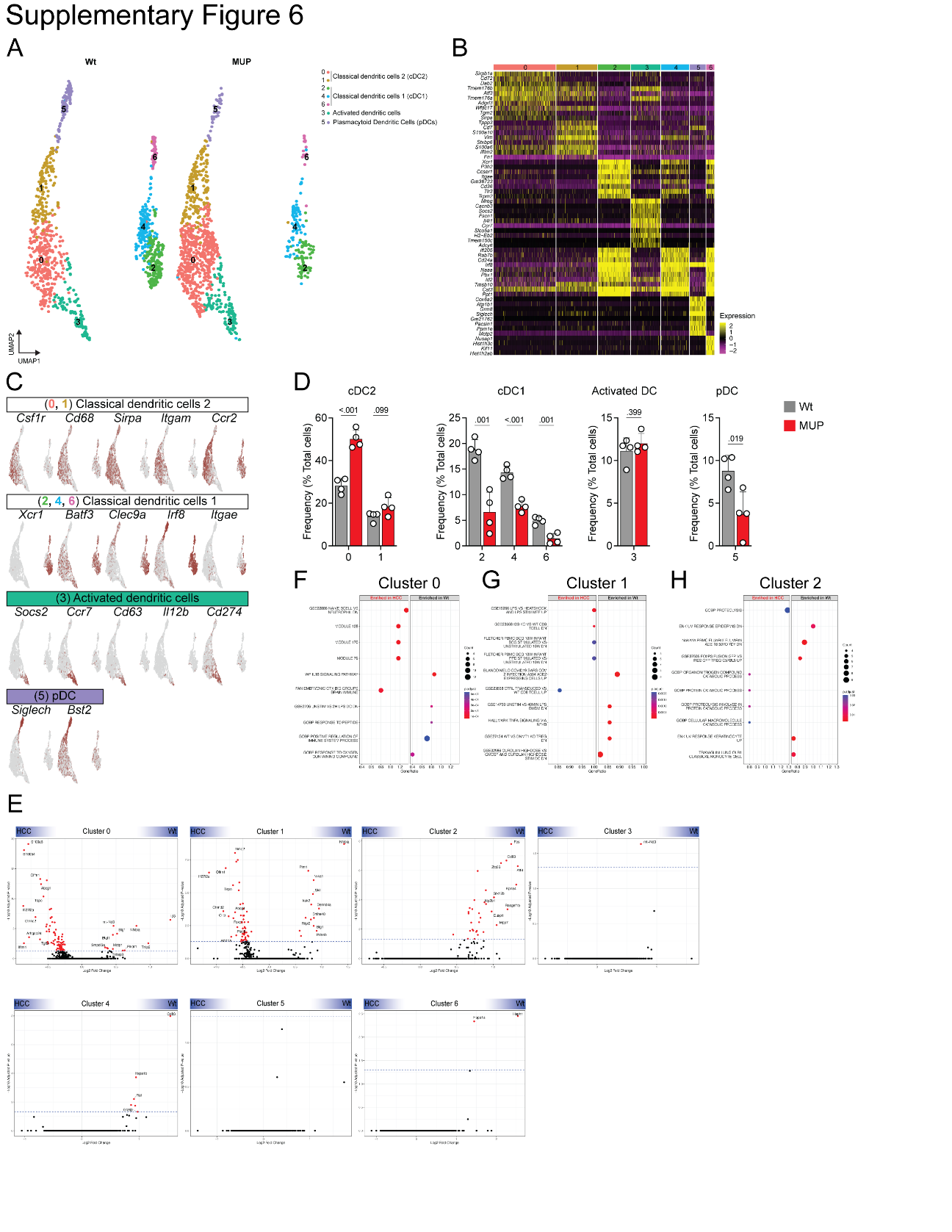

Supplement: Supplementary file 1 [file hc9-9-e0668-s001.docx]
